# Book Reviews

**Published:** 1898-03

**Authors:** 


### Book Reviews.

THE CARE AND FEEDING OF CHILDREN.— A Catechism for the Use of Mothers and Children's Nurses. By L. Emmett Holt, M. D. Second Edition. Revised and Enlarged. New York, D. Appleton & Co., 1898.

This little book contains in the form of questions and answers a great deal of practical information as to the care and feeding of children, which will be found of value to those having the charge of children as well as to physicians.

The questions embrace a great variety of subjects, as bathing, clothing, nursing, weaning, mother's milk with the composition and preparation of various substitutes with directions for using them. A number of food formulæ are given, and directions for making special preparations.

A part of the book is devoted to the general management of babies and young children, what to be done and what to be avoided.

It is entirely within the range of every intelligent mother and may be put in their hands with advantage.

DISEASES OF THE STOMACH.—Their Special Pathology, Diagnosis, and Treatment, Analysis of Stomach Contents, Dietetics, Surgery of the Stomach, etc. In Three Parts. By John C. Hemmeter, M. B., Ph. D., M. D. Clinical Professor of Medicine, Baltimore Medical College, etc. With many Original Illustrations, a Number of Which are in Colors, and a Lithographic Frontispiece. Published by P. Blakiston Son & Co, Philadelphia, 1897. Price $6 00.

This is an entirely new contribution to the pathology and treatment of organic diseases of the stomach, and is by far the most comprehensive and useful work that has yet appeared upon this important subject. Many new methods of diagnosis are given, and the work of American clinicians in this special department has been carefully considered. The chief effort of the author has been to furnish the general practitioner with a work from which he can readily acquaint himself with all that has been done in this important branch of medicine, to fit himself to make examinations, to take advantage of new methods of diagnosis, and to treat this very difficult class of diseases rationally and successfully. The subject-matter has been treated sytematically, and concisely, giving first, the special anatomy and physiology of the digestive organs, methods of diagnosis and general therapy, including dietetics, following this by a methodical discussion of the various diseases affecting the stomach, with their symptomatology, diagnosis, prognosis, pathology, and treatment. The work is well illustrated throughout, a number of which are original. The publishers have executed their work in a most creditable manner, as is their custom, and taken all in all, this is in our opinion the best work of its kind in print.

A TREATISE ON DISEASES OF WOMEN.—For the use of Students and Practitioners of Medicine. By Alexander J. C. Skene. M.D., L.L D. Third Edition. Revised and Enlarged, with 290 Engravings and 4 plates in color. D. Appleton & Co., New York, 1898.

The third edition of this well-known and highly valued work gives every evidence of being fully abreast of all the advancements made in this progressive province of medicine. When this is said, a great deal is said, for no department of medicine has exhibited more feverish activity than gynæcology, especially the surgical phase of it. To keep in pace with its constant mutations, is sufficient to make the stoutest heart shrink from the task of sifting and collecting from the vast amount of material that which is worthy of being preserved and given to students, with the stamp of the author's approval.

The present volume contains considerable additional matter as well as laudable attempts at simplification of the various methods of surgical treatment. The subject of rational treatment of diseases of the urethra and bladder is entered into at length, including clear directions as to the use of the endoscope and cystoscope.

The author describes and recommends his method of controlling hemorrhage without ligature by means of the electric hemostatic forceps. This is in the shape of an ordinary compression forceps and is arranged in such a manner that an electric current passes through the handles of the instrument and is concentrated upon the inner surfaces of the jaw of the forceps. The bleeding vessel or mass of tissue is grasped and a current sufficient to dry the parts is passed through. The author claims that hemorrhage is controlled, and that the ligature, always a source of uneasiness, can be dispensed with.

The book contains nearly a thousand pages and has numerous accurate illustrations.

